# Title: “Labels Matter: Is it stress or is it Trauma?”

**DOI:** 10.1038/s41398-021-01514-4

**Published:** 2021-07-10

**Authors:** Gal Richter-Levin, Carmen Sandi

**Affiliations:** 1 Sagol Department of Neurobiology, University of Haifa, Haifa, Israel; 2 Psychology Department, University of Haifa, Haifa, Israel; 3 The Integrated Brain and Behavior Research Center (IBBR), University of Haifa, Haifa, Israel; 4 Brain Mind Institute, Ecole Polytechnique Fédérale de Lausanne (EPFL), Lausanne, Switzerland

**Keywords:** Neuroscience, Psychology, Drug discovery

## Abstract

In neuroscience, the term ‘Stress’ has a negative connotation because of its potential to trigger or exacerbate psychopathologies. Yet in the face of exposure to stress, the more common reaction to stress is resilience, indicating that resilience is the rule and stress-related pathology the exception. This is critical because neural mechanisms associated with stress-related psychopathology are expected to differ significantly from those associated with resilience.

Research labels and terminology affect research directions, conclusions drawn from the results, and the way we think about a topic, while choice of labels is often influenced by biases and hidden assumptions. It is therefore important to adopt a terminology that differentiates between stress conditions, leading to different outcomes.

Here, we propose to conceptually associate the term ‘stress’/‘stressful experience’ with ‘stress resilience’, while restricting the use of the term ‘trauma’ only in reference to exposures that lead to pathology. We acknowledge that there are as yet no ideal ways for addressing the murkiness of the border between stressful and traumatic experiences. Yet ignoring these differences hampers our ability to elucidate the mechanisms of trauma-related pathologies on the one hand, and of stress resilience on the other. Accordingly, we discuss how to translate such conceptual terminology into research practice.

## Introduction

Our choice of labels and metaphors for the information we observe in the world impacts the way we process and understand it [1]. This is true also in science, where choice of terminology is often influenced by hidden assumptions and predispositions, which in turn may affect not only research directions, but also the conclusions drawn from the results [1]. Taking the time, every now and then, to reflect on customary terminology and on the potential benefit of considering alternative terminology is important for any field of research. It is certainly relevant for the field of the neurobiology of stress to reflect and consider hidden biases related with the use of the term *Stress* [2, 3].

*Stress* has a negative connotation. Particularly because of its known potential to trigger or exacerbate pathologies [4–7]. Yet both humans and animals demonstrate surprisingly high resilience to it [8–10], indicating that resilience is the rule and stress-related pathology the exception. Indeed, the physiological stress response has been selected throughout evolution as an important survival reaction [11, 12]. Thus, it seems likely that the neural mechanisms associated with trauma-related psychopathology are different from the neural mechanisms that are triggered by stressful experiences that do not lead to trauma-related psychopathology [13]. This likely distinction, emphasizes the importance of differentiating between the terms ‘*stress*’ and ‘*trauma’*. In order to differentiate between neural mechanisms that are associated with the pathology and those actually associated with stress resilience, it is critical to be able to differentiate between individuals who were exposed to a significant emotional challenge but did not develop pathology and those who did.

### Understanding the stress-response: from simplistic text-book descriptions to a complex integrative view

The stress-response system is often described as a combined neuroendocrine response, with a bias toward emphasizing its endocrinological arm: an encounter with a stressful challenge activates simultaneously the sympathetic nervous system (SNS) and the hypothalamus–pituitary–adrenal (HPA) axis. This response, often referred to as ‘*the’ stress response system*, comprises two stages: first, a fast response, in which the SNS is activated to trigger a myriad of peripheral actions, both through direct action of sympathetic nerves and through the release of adrenaline from the core of the adrenal gland. Second, a slower response, known as HPA axis response, in which the release of corticotropin-releasing hormone (CRH) from the paraventricular nucleus of the hypothalamus induces the release of adrenocorticotropic hormone (ACTH) from the anterior pituitary gland, which in turn triggers the release of cortisol (or corticosterone) from the adrenal cortex.

While these textbook descriptions are correct, they often present a misleading oversimplification of what is taking place and miss the essence of the stress response. Too frequently, cortisol is referred to as the ‘stress hormone’, implying that it is the most significant aspect of the stress response, and reflecting literature’s overemphasis of its role in the stress response [14]. Yet, while cortisol is indeed secreted during stress, it takes over ten-to-twenty minutes for cortisol blood levels to rise to their stress-related peak [15]. This is significant since many highly stressful situations, such as predator–prey encounters, normally last only a few minutes, as any National Geographic documentary fan well knows.

In fact, cortisol has a dual role in the stress response: in addition to mobilizing resources to cope with a current stressor, once the threat is over, cortisol is actually responsible for bringing the stress response to its end, via its inhibitory feedback effect on the hypothalamus and pituitary gland [16, 17]. If the threat is still present, the role of cortisol is to shift the stress response into a longer-period response, balancing between the emergency needs for energy for physical responses and the need to maintain the ability to respond for a longer period of time. Furthermore, cortisol is not secreted only in response to stress and is not triggered only by negative challenges or adverse experiences. Pleasurable activities, such as sports and sex, also lead to increased secretion of cortisol [15, 18].

More recently, there is growing attention to the energy component of the stress response [19, 20]. Cortisol’s role as a metabolic hormone is emphasized in this context, because of its effects on increasing blood glucose levels and its capacity to eventually directly affect mitochondrial function [19–22]. Indeed, an important part of the stress response is metabolic control, as increases in glucocorticoid levels can lead to increases in glucose and modulate adiponectin levels [22–26]. Furthermore, stress and trauma-related psychopathologies, such as post-traumatic stress disorder (PTSD), or stress-induced mood disorders, are known to have high comorbidity with metabolic dysregulation and metabolic syndromes [19, 27, 28].

Therefore, the stress response emerges nowadays as a complex and integrative process that involves, among others, neuronal–neuroendocrine-immune interactions [19, 23, 27, 29–34] and these interactions are also influenced by an individual’s genetic background [35–39].

### The challenge of defining a *stress response*

Attempts to define the concept of *Stress* frequently fail to encompass its complexity [14, 15, 40, 41]. Hans Selye, often credited as the modern-day father of stress research, defined stress in 1936 as “the nonspecific response of the body to any demand for change” [42]. This definition is clearly too general and circumscribes the term *stress response* to the body’s response to *a stressor*. Yet more than eighty years after Selye’s original definition [42], a clear definition of the *stress response* remains elusive. One important reason for this is that any response to any environmental stimulus involves some physiological reaction. Intuitively, when researchers refer to a *response* to *a stressor*, they refer to a response to a challenging event, which would seem to require a significant reaction. Yet at what stage does a response start to fall into the category of a *stress response*? This is where the lines begin to blur. To make matters worse, many of the physiological reactions that could form part of a *stress response* could just as well be components of responses to much milder or positive challenges that would not fall into the category of *a stressor*.

Attempts to address this difficulty in definition brought up the terms *Allostasis* and *Allostatic Load* [31]. *Allostasis* refers to the mechanisms employed to achieve stability through change in response to constantly changing social and physical environments [43]. In many cases, *Allostasis* involves *adaptive plasticity*, i.e., lasting neurobiological, endocrine, or immune-response alterations, designed to adjust the body physiology to the new conditions [31]. The term *Allostatic Load* was later proposed to refer to the long-term consequences of activating allostatic mechanisms [44], and to emphasize that, while a brief activation of allostatic mechanisms in response to environmental challenges may be adaptive, prolonged activation, in response to chronic challenges, is likely to lead to pathological outcomes [45]. Indeed, the term *allostatic overload* was introduced in order to emphasize that the same stress response, when activated for long periods of time, may come with a cost to the organism and establish the foundation of various metabolic, cardiovascular, neurological and mental diseases [34, 46, 47]. Recently, there have been attempts to create a diagnostic scale of allostatic overload that would help guiding treatment [48].

### The subjective nature of the experience

When people describe a stressful experience, the intensity of the stress is often related to the characteristics of the stressor: “…it was a terrible car accident…,” “…they experienced an awful earthquake…,” “…she survived a horrible terrorist attack…”. Indeed, a serious car accident, which involves people injured, is more likely to lead to the development of psychopathological symptoms than a mild car accident, in which no one was hurt. The same principle is used for designing animal experiments. For example, using 0.4 mA as a mild-intensity foot-shock experience, and 1 mA as a severe stressful experience [49, 50]. On average, it can be expected that the stress-response measurements of the group that received 1-mA foot shock would be higher than those of the group receiving 0.4 mA.

Yet the characteristics of the *stressor* by themselves do not define the intensity of the individual stress experience, they only contribute to it [51, 52]. This is a critical aspect of stress, and it makes it impossible to define a stress experience by using only the parameters of the stressor. The impact of exposure to a stressor is a combination of the parameters of the stressor, the characteristics of the individual’s physiological machinery, and the subjective way the stressor is perceived by the individual [53–56]. The subjective nature and the huge individual variability observed following exposure to a stressor is one of the defining hallmarks of the stress response [13, 54, 57–59]. For example, the lifetime prevalence of exposure to severely stressful events like combat, accidents, natural disasters, assault, or rape is as high as 75–80% [60, 61]. If the characteristics of the stressor were the major factor in defining the outcome, we would expect that the prevalence of trauma-related disorders, such as PTSD, would reflect this high percentage of exposure to severely stressful events. Yet only about 10–20% of the population exposed to these types of stressors will suffer from clinically relevant PTSD [60, 62–65]. In other words, regardless of the characteristics of the trauma, only a minority of the exposed individuals eventually develop pathology. This signifies the importance of both the neurobiological constitution of individuals in producing stress responses and the subjective nature of the impact of stress and trauma, i.e., the same stressful event may lead to very different responses in different individuals. This is true for both humans and animals. It is now becoming clear that stressful experiences cannot be defined solely by the conditions to which an individual was exposed, because the eventual emotional experience of these conditions will also greatly depend on the combination of their physiological constitution and their subjective perception of the event by the individual—the *perceived experience*.

The realization of the critical role of the subjectivity of stress experiences represents a significant challenge to the neurobiology of stress field. The scientific method, which often relies on statistical analyses of averages of treatment groups, is not very efficient in analyzing subjective experiences. Animal models, which are the main window to understanding physiological and neurobiological processes underlying behavior and psychopathologies, are even less geared to do so.

### The need for a stronger conceptual differentiation between *Stress* and *Trauma*

Our claim is that the use of the term *Stress* in a nondistinctive way to describe emotional experiences anywhere on the range from mild to severe, contributes to the difficulty in addressing the subjective nature of the response to emotional, stressful, or traumatic experiences.

Not every challenge should come under the umbrella of the term *Stress*. We propose to refer to three distinct **conceptual** levels of emotional experiences, which will be distinguished from one another according to three parameters: the level of emotional reaction associated with the experience, the scale of induced plasticity from the experience, and, most importantly, the experience’s impact on the ability or inability of the individual experiencing it to cope later on with daily challenges.

The following are the three distinct conceptual levels we propose:

### Arousing experience

An experience that activates a detectable emotional reaction and may affect the immediate choice of behavioral response, but does not lead to substantial long-term consequences in physiological systems, i.e., it does not lead to physiological plasticity. Following an arousing emotional experience, physiological systems generally return to their former baseline.

### Stressful experience

An emotionally significant experience that activates a substantial emotional response—a response that not only acutely changes the choice of an immediate behavioral response, but also induces lasting alterations that have the potential of changing the response of the individual to a variety of future experiences (***metaplasticity***—plasticity of plasticity) [66, 67]. Those alterations are not pathological, however, as they remain within the coping abilities of the individual [15]. As a result, their **functional capacity**, understood as the degree to which the individuals can adapt with normative responses to future challenges, is not affected.

### Traumatic experience

An experience that activates a robust emotional response, which not only acutely changes the choice of a behavioral response, but also induces lasting alterations that would change the response of the individual to a variety of future experiences. Critically, those alterations are pathological, compromising functional capacity, i.e., the ability of the individual to cope later on with daily challenges. In other words, it can be said that the experience induces ***pathological metaplasticity***.

**Fig. 1:**
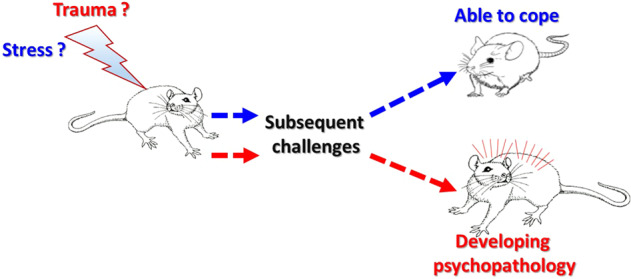
Schematic illustration addressing the need for a conceptual terminology to allow the distinction between Stress and Trauma. As of now, there are no ideal ways for effectively addressing the subjective nature of the response to stress, the murkiness of the border between stressful and traumatic experiences, and the complexity of individual variability. Nevertheless, we propose to conceptually associate the term ‘stress’/‘stressful experience’ with ‘stress resilience’ (the ability to be exposed to a stressful experience and cope with it), while restricting the use of the term ‘trauma’ only in reference to exposures that lead to pathology. In accordance with that, additional tools and approaches should be developed, making it more practical to establish the distinction between Stress and Trauma. These would enable to more effectively dissociate neural mechanisms underlying coping with stress from those mechanisms underlying failure to cope, which lead to the development of trauma-related psychopathologies.

It should be emphasized that we propose these definitions as **conceptual** distinctions. They obviously leave much to be more precisely defined: what should be considered as a ‘significant’ or a ‘robust’ emotional response? What should be considered as a ‘substantial level of plasticity’? How do we define healthy functional capacity? What should be considered a pathological outcome? When does an experience transform from ‘stressful’ to ‘traumatic’? etc. For both humans and animals, these are all challenging concepts to define. We will propose how to start translating these concepts into practice below, but regardless of whether and how precisely we can draw the dividing lines, it is critical to ***conceptually*** distinguish between these notions (Fig. 1). Particularly, it is important to distinguish between *stressful* and *traumatic* experiences. As mentioned above, our choice of terminology shapes our way of thinking about scientific challenges, and the way we address them [1–3]. The questions indicated above, such as how to define a ‘substantial level of plasticity’ or ‘healthy functional capacity’, would not have been considered if not for such conceptual distinction.

In our view, the use of the term *Stress* for both stressful and traumatic experiences has hampered the field’s ability to dissociate between two distinctive mechanisms. People who have undergone a severe experience, may change their behavior in significant ways and, if examined, will exhibit neuronal alterations at different levels of analysis. Yet many of them will continue with what is considered regular daily life and a satisfactory level of day-to-day functional capacity. What this means is that these people do not suffer from a pathology. Somehow, they have managed to cope with the experience. It is, therefore, important to clearly distinguish these individuals’ experience from that of others, who, as a result of being exposed to a similar experience, have lost a significant part of their previous neurobehavioral functional capacities. Accordingly, the same experience affects neural mechanisms differently and induces a different level of plasticity in the two cases. Similarly, in animal studies, we should be able to distinguish between alterations in behavior and physiology that lead to a significant compromise of the animal’s functional capacity and those alterations that do not [68, 69]. Lumping together the results from both affected and coping individuals and averaging them out would mask any possibility to identify the mechanisms of stress vulnerability, pathology or stress resilience.

The proposed conceptual definitions emphasize the **consequences** of exposure to an experience. This raises a problem when referring to *resilience* or *vulnerability* to traumatic experiences, because resilience and vulnerability refer to characteristics that are assumed to be present prior to the exposure and influence the exposure outcome [70–72]. Below, we suggest a way to use the concepts of resilience and vulnerability in the context of the above-proposed dissociation between *stress* and *trauma*.

### Translating the conceptual terminology into research practice

The conceptual dissociation between *stress* and *trauma* suggested above raises practical problems for studies of stress and trauma. It becomes imperative to consider when a stress experience should be defined as traumatic and how to distinguish between a *stressful* experience and a *traumatic* one. The main challenge arises from the subjectivity of the response: the very same experience could be experienced as *stressful* by one individual (i.e., significantly challenging but nevertheless still within the coping abilities of that individual), and as *traumatic* by another one. Currently, there is no convenient way to define and quantify the ‘perceived experience’. Therefore, in order to address this issue, we propose that animal studies undertaken within this field (and to some extent, also human studies) categorize parameters in the following five complementary aspects:

A. The choice of the stress/trauma protocol
B. Considering modulating factors
C. The choice of tests to assess functional capacity
D. The data analysis approach
E. The discussion of the results

We will now discuss each of these aspects in more detail.

## The choice of the stress/trauma protocol

As discussed above, the subjective nature of the stress experience leaves no possibility of clearly defining a protocol as either *stressful* or *traumatic*. Yet taking the conceptual distinction between the two into consideration is instrumental for effective progress in understanding the psychological, neurobiological, and pathological aspects of stress and trauma. When planning a study, already at the first step, defining whether the focus will be on *stress* or *trauma* in their suggested definitions here, will make a difference in the selection of examined populations and the experimental procedures employed.

Although a clear distinction between a *stressful* or *traumatic* experience cannot be made based only on the exposure protocol (i.e., on the characteristics of the stressor/s), it is still true that the milder the protocol, the more likely it is that more individuals will experience it just as *stressful*, and the more intense it is, the greater proportion of tested individuals will experience it as *traumatic*.

### In fact, several factors are known to make an experience more intense

#### Physical intensity of the experience

Although the physical intensity of the triggering challenge alone cannot define the individual’s outcome, it is in correlation with the outcome. The stronger the electric shock or the colder the water individuals are exposed to, the more likely the incident to be experienced as *traumatic* [49, 73]. Another factor that may add to the intensity of the experience is the chronicity of a stressor [5, 18, 45, 47, 69].

#### Level of predictability

Predictability is known to be a protective factor against demanding challenges [74, 75]. The less prepared the individual is for the experience, the more surprising the experience, the greater the impact it will have [76–78].

#### Level of controllability

Probably the most influential factor to determine the impact on the individual of exposure to challenging experiences is the level of controllability over the exposure. It has been elegantly and convincingly demonstrated that the precise same physical exposure could be *stressful* under controllable conditions but *traumatic* under uncontrollable conditions [79–83].

Taking the above into consideration, when the research goal is specifically to study trauma mechanisms, it is important to use a stressor or a combination of conditions that are more likely to be experienced as trauma by a larger percentage of the exposed population.

It is important to note here that an animal model of PTSD presents an ethical challenge: on the one hand, a key ethical principle is to reduce unnecessary suffering of the animals, but on the other hand, another important principle is to perform experiments in a way that would maximize the probability of gaining the searched for understanding. Aiming to understand the mechanisms of trauma and related pathologies requires exposure of animals to what would most likely be perceived as trauma. Exposing them to a mild stressor, which is not likely to be perceived as traumatic, may be more acceptable by the ethical committees, but would compromise the relevance of the experiment to trauma-related psychopathologies. Accordingly, we would like to add an important warning here that choosing a “less stressful” protocol in order to get the ethical committee consensus or being enforced by the committee to choose a “less stressful protocol” for ethical reasons may be counteractive, as this approach often leads to the usage of animal models devoid of any translational value, causing a much greater ethical problem.

## Considering predisposition factors

Several modulating or predisposition factors were found to influence both the magnitude of physiological reaction and the subjective experience of an exposure to a stressor. These may either be used in order to aim for an outcome of *stress* or *trauma*, when planning an experiment, and when choosing the study population, or should be taken into consideration when discussing the outcome of a study.

### Mostly, three predisposing factors are considered

#### Genetic predisposition

It is widely accepted that there are genetic influences on the development of PTSD [84, 85], although available data suggest that stress-related disorders are highly polygenic and the relations to specific genes are complex [84–86]. Several genes, such as glucocorticoid-induced leucine zipper (GILZ), a transcription factor encoded by the gene Tsc22d3 on the X chromosome, the serotonin transporter gene (SLC6A4), or pituitary adenylate cyclase-activating polypeptide (PACAP), are suggested as being more influentially associated with the risk of developing stress-related psychopathologies [86, 87]. More often, gene polymorphisms were found to be of influence only when associated with another predisposing factor—childhood adversities [87, 88].

#### Previous life experiences

It has been demonstrated that exposure to harsh experiences at critical developmental periods could serve as a risk factor for the reaction to subsequent stressful experiences, transforming a *stressful* experience into a *traumatic* one for the individual. Early-life adversities [89–93], childhood adversities [94, 95], and adolescence adversities [96–98] have been indicated as risk factors that intensify the impact of exposure to a stressful experience in adulthood. Proximal factors, such as sleep disturbances, appear to act as well as risk factors [99–101]. Pre-exposure to such adversities would increase the probability of a stressful experience later in life to be *traumatic*, i.e., to lead to high levels of pathological symptoms and to significantly compromise the ability of the individual to cope with day-to-day challenges. It should be noted though that some forms of pre-exposure to stressors may actually result in the development of resilience, if that pre-disposing experience leads to the development of coping strategies that support coping with stressors later in life [102, 103]. While this further complicates translating the dissociation between ‘Stress’ and ‘Trauma’ into practicality, conceptually, it actually strengthens the need to dissociate between the two. In order to correctly dissociate and study early-life experiences that lead to vulnerability or to resilience, one needs to define whether they increased or not the likelihood of developing pathology. In order to faithfully answer that, it is critical to be able to dissociate between individuals who eventually did or did not develop pathology.

#### Level of social support leading to, during and following the exposure

Studies have shown that the lack of social support leading to, during and following the exposure to a stressful experience, could influence to a large extent the response of the individual to the exposure [104–108].

## The choice of tests to assess functional capacity

The newly proposed definitions for *stress* and *trauma* require typifying each participant and determining whether or not their *functional capacity* was impaired in a significant way. *Functional capacity* is not, as such, a well-defined or quantitative term. Further studies are required in order to develop widely accepted tests. Yet one principle has already emerged from the literature: in both human and animal studies, a battery of tests that examines a wide range of behavioral domains is required. A single test can identify a specific alteration but cannot depict the true impact of the experience on an individual. A battery of tests is required also in order to be able to identify sub-populations of individuals who may be affected by exposure to the challenging circumstances in different ways. For example, the exposure to a traumatic experience may lead to a different pathological profile (e.g., more anxious or more depressive) in subgroups of exposed individuals [109–112] or to different clusters of symptoms, as it has been shown, for example, for males and females [95, 113–116].

It is too early to propose employing an agreed-upon, unified set of tests that would serve as an accepted standard for *functional capacity*. Different labs are still at the important exploratory phase, examining the efficacy of different test batteries. Furthermore, it is likely that different standardized batteries of tests would be required for different behavioral predispositions or pathological clustering. However, the eventual goal should probably be to standardize tests, at least in the context of specific pathologies, and we make a call here for a future focus on this endeavor.

Importantly, as indicated above, findings from both humans and animals indicate that males and females are differently sensitive to different stressors and may be affected in different behavioral domains [117–123]. Thus, when choosing the exposure protocol and the test batteries to be used, it is critical to take sex differences into consideration.

## The data analysis approach

Whether an event will be experienced as *stressful* or *traumatic* cannot be delineated from the characteristics of the stressor. As indicated above, there is high individual variability in responses to the same experience. This is true even for rodent-inbred lines, which are supposed to be genetically identical, due to experience-induced plasticity and epigenetic processes [123–132]. Typically, individual differences are concealed by the use of group averages, by increasing the sample size, and even by excluding outliers [133]. Yet with respect to stress and trauma, individual variability and outliers are at the essence of the phenomena. It is crucial to dissociate and differentiate between individual clusters of responses in order to associate specific biological mechanisms to precise behavioral responses. For example, in the case of PTSD, if only about 10–20% of the exposed population is expected to develop the disorder, working with the averaged data of the exposed group would clearly hamper the possibility of identifying biological mechanisms associated with the pathology.

It is therefore critical to shift away from working with group averages and move toward more individualized analysis approaches. Some years ago, Cohen et al [134, 135] presented the *cutoff behavioral criteria (CBC)* analysis approach to differentiate between *maladaptive* and *well-adaptive* individuals. While the CBC analysis approach was found to be very productive, yielding a series of highly relevant findings [136], which could not have been identified otherwise, it is different from the way diagnosis is done in humans. While human studies take into consideration responses in nonchallenged individuals, the *CBC* analysis focuses only on exposed individuals, comparing the performance only among them. More recently, we have developed a variation of the *CBC* in rodents, termed *behavioral profiling*, which is an analysis approach based on referring to the performance of a control, nonexposed group as defining normal behavior, and assessing deviation from that “norm” [13, 137, 138]. Importantly, control and exposed animals are examined on a battery of tests, aiming to test their *functional capacity* and to assess their coping abilities. Employing this analysis approach enabled us to demonstrate key neurobiological differences between animals that were exposed to the traumatic experience and developed symptoms (exposed-affected) and those that were exposed to the same experience but did not develop significant symptoms (exposed–nonaffected) [137–139], further verifying the functional significance of such a distinction. This approach is in good alignment with the human population in which there are people who develop PTSD (susceptible) and others who do not (resilient) after trauma exposure. In addition, differentiating between exposed–affected and exposed–nonaffected individuals enables differentiating between neural mechanisms that are associated with the pathology and those actually associated with stress resilience [137, 139]. Moreover, other studies in the literature have succeeded to predict susceptibility to develop a PTSD-like phenotype on the long term, by characterizing animals’ exploratory responses in a novel environment [140] or measuring their changes in startle reactivity [141] shortly after trauma exposure. This line of work aligns well with evidence in humans indicating that a Clinician-Administered PTSD Scale for DSM-IV (CAPS) given within 60 days of trauma exposure is highly predictive of the degree and persistence of PTSD symptoms at later stages [142].

Furthermore, other approaches go beyond the postexposure characterization of animals (i.e., ‘sequalae factors’) to identify ‘susceptibility factors’ pre-existing before trauma [143]. For example, the ‘Revealing Individual Susceptibility to a PTSD-like Phenotype’ model [144] assesses susceptibility to trauma (segregating susceptible, resilient, and intermediate animals) according to rats’ responsiveness (i.e., startle and anxiety-like responses) as assessed a few days after the first encounter of a mild stressor. These approaches may help identifying susceptible individuals a priori and guide neurobiological studies with the potential of paving the way toward the development of preventive treatments.

## The discussion of the results

Even before changing anything in the experimental protocols, we believe that the proposed definitions of *Stress* and *Trauma* are important for a more productive discussion over existing findings. The confusion arising from the use of the term *stress* for describing a wide range of different experiences and the responses to them has introduced great difficulties in comparing the findings, discussing them and drawing conclusions from them in a comprehensive way.

Adopting the proposed terminology and aiming to plan and conduct experiments along the line of those definitions will enhance discussion and promote reaching more well-based conclusions in the future.

### Vulnerability and resilience versus pathology and no pathology

The proposed conceptual definitions of *stress* and *trauma* are based on the eventual outcome of the exposure, namely, whether the individual could maintain its *functional capacity* or failed to keep effective coping when facing future life events. This terminology is effective when examining the outcome of an exposure. Yet, difficulty arises when attempting to associate them with questions regarding *vulnerability* versus *resilience* (and we are back to the importance of semantic definitions). Often, the terms *vulnerability* and *resilience* are used in a predictive manner, to describe qualities that would only be manifested if and when individuals will encounter a challenging experience [145]. The assumption behind the use of these terms is that vulnerable or resilient individuals are already different prior to the potential encounter, in a way that would enable the resilient, but not the vulnerable individual, to cope with the challenge if and when it arises [146, 147].

It is important to note that even when referring to *vulnerability* and *resilience* as qualities to be measured prior to or without exposure to any challenge, the concept is a predictive concept, referring to currently present mechanisms that would come to action, or fail to come to action in the face of future exposure. In fact, *vulnerability* and *resilience* require identifying biomarkers that can predict the coping abilities of the individual. In that sense, the conceptual definitions proposed here for the dissociation between *stress* and *trauma* still hold, and they help to identify the need for predictive biomarkers.

As of now, there are no actual biomarkers that could predict *vulnerability* or *resilience* in an effective way. However, employing the *Behavioral Profiling* analysis approach, which enables to differentiate between exposed–affected and exposed–nonaffected individuals, it is possible to design studies that would collect samples prior to the exposure and analyze them in light of the eventual outcome, thus associating them to *vulnerability* or *resilience*. In any case, planning such experiments requires dissociating between *stressful* or *traumatic* outcome, if to reflect any functional significance to identified biomarkers.

### Recovery

A final point to discuss is the fact that PTSD in humans is considered as a failure to follow the normative trajectory of recovery (e.g., in terms of physiological, cognitive, or behavioral symptoms) after exposure to a traumatic event [148]. Recent evidence indicates that some individuals may take long periods of time to recover from PTSD [149] and, therefore, animal studies may need to define the range of testing that would allow characterizing deficiencies in remote symptoms, e.g., [150].

### Summary

We acknowledge that there are as yet no ideal ways for effectively addressing the subjective nature of response, the murkiness of the border between *stressful* and *traumatic* experiences, and the complexity of individual variability. However, ignoring these challenges hampers our ability to elucidate the mechanism of trauma-related pathologies, and of resilience. There is already sufficient evidence to indicate that pathological metaplasticity, which is expected to take place in the brains of individuals who develop behavioral pathologies following exposure to a traumatic event, would be very different from the type of meta-plasticity that could be found in the brains of those individuals who do not experience the event as traumatic, and do not develop pathology. We have proposed here to start focusing on differentiating between ‘*stress*’ and ‘*trauma*’ and conceptualizing the semantic difference between them. Adopting the conception, terminology and differentiation between the concepts of *Stress* and *Trauma*, is a first step to inspire planning and conduct of both human and animal studies in accordance with this conception. Likewise, the evaluation of the results and discussion of their most likely interpretation will benefit from the clarity provided by these distinctions. Furthermore, such semantics will encourage the search for more suitable research and analysis tools.

Recently, analysis approaches that put more emphasis on the behavioral profiling triggered by exposure to challenging events by focusing on individuals rather than on group averages have been developed and prove to be very effective and relevant (e.g., [13]). In the future, additional tools and approaches should be developed to allow making the distinction between *Stress* and *Trauma* easier to establish. Consequently, neural mechanisms underlying coping with stress could be more effectively dissociated from those mechanisms underlying failure to cope and the development of trauma-related psychopathologies. Ultimately, these dissociations should allow refining our findings and, thus, facilitating the development of more effective treatments for trauma-related psychopathologies.
